# Long-Term Effect of Mechanical Thrombectomy in Stroke Patients According to Advanced Imaging Characteristics.

**DOI:** 10.1007/s00062-023-01337-4

**Published:** 2023-08-29

**Authors:** Morin Beyeler, Fabienne Pohle, Loris Weber, Madlaine Mueller, Christoph C. Kurmann, Adnan Mujanovic, Leander Clénin, Eike Immo Piechowiak, Thomas Raphael Meinel, Philipp Bücke, Simon Jung, David Seiffge, Sara M. Pilgram-Pastor, Tomas Dobrocky, Marcel Arnold, Jan Gralla, Urs Fischer, Pasquale Mordasini, Johannes Kaesmacher

**Affiliations:** 1 Department of Neurology, Inselspital, Bern University Hospital, University of Bern, Freiburgstrasse 18, 3010 Bern, Switzerland; 2 Department of Diagnostic and Interventional Neuroradiology, Inselspital, Bern University Hospital, University of Bern, Freiburgstrasse 8, 3010 Bern, Switzerland; 3 Neurology Department, University Hospital of Basel, University of Basel, Basel, Switzerland

**Keywords:** Mechanical thrombectomy, RAPID software, Ischemic core volume, Perfusion mismatch, Hypoperfusion intensity ratio, Long-term outcomes

## Abstract

**Purpose:**

Data on long-term effect of mechanical thrombectomy (MT) in patients with large ischemic cores (≥ 70 ml) are scarce. Our study aimed to assess the long-term outcomes in MT-patients according to baseline advanced imaging parameters.

**Methods:**

We performed a single-centre retrospective cohort study of stroke patients receiving MT between January 1, 2010 and December 31, 2018. We assessed baseline imaging to determine core and mismatch volumes and hypoperfusion intensity ratio (with low ratio reflecting good collateral status) using RAPID automated post-processing software. Main outcomes were cross-sectional long-term mortality, functional outcome and quality of life by May 2020. Analysis were stratified by the final reperfusion status.

**Results:**

In total 519 patients were included of whom 288 (55.5%) have deceased at follow-up (median follow-up time 28 months, interquartile range 1–55). Successful reperfusion was associated with lower long-term mortality in patients with ischemic core volumes ≥ 70 ml (adjusted hazard ratio (aHR) 0.20; 95% confidence interval (95% CI) 0.10–0.44) and ≥ 100 ml (aHR 0.26; 95% CI 0.08–0.87). The effect of successful reperfusion on long-term mortality was significant only in the presence of relevant mismatch (aHR 0.17; 95% CI 0.01–0.44). Increasing reperfusion grade was associated with a higher rate of favorable outcomes (mRS 0–3) also in patients with ischemic core volume ≥ 70 ml (aOR 3.58, 95% CI 1.64–7.83).

**Conclusion:**

Our study demonstrated a sustainable benefit of better reperfusion status in patients with large ischemic core volumes. Our results suggest that patient deselection based on large ischemic cores alone is not advisable.

**Supplementary Information:**

The online version of this article (10.1007/s00062-023-01337-4) contains supplementary material, which is available to authorized users.

## Introduction

Mechanical thrombectomy (MT) is the standard of care for large vessel occlusion strokes and advanced imaging (CT perfusion or MRI-based diffusion and perfusion imaging) assessments have allowed for expanding the indication group for this treatment [1–3]. However, the radiological selection criteria remain controversial in certain situations, especially in the case of large ischemic core [4]. According to two trials on thrombolysis, an arbitrary chosen ischemic core volume of 70 ml is commonly used as the cut-off for large infarcts and was defined as the upper limit regarding MT’s indication [5, 6]. In addition to the ischemic core volume itself, the mismatch between the core and the tissue at risk (commonly called penumbra) plays a pivotal role in patient selection for MT [4, 7]. Hypoperfusion intensity ratio (HIR) is used to assess collateral status with low HIR being associated with robust collaterals and described as a potential selection factor for thrombectomy eligibility and a predictor of favorable outcome [8, 9]. Despite residual uncertainties regarding the benefit of MT beyond 6 h from symptom onset in large ischemic cores, the short-term benefit of MT in this population after selection by advanced imaging criteria are well known [9–13]. In contrast, the long-term effects of MT, especially based on advanced imaging criteria, remain poorly described [14]. This study aimed to evaluate the influence of reperfusion status on the long-term outcomes (mortality, functional outcomes and quality of life) according to strata of baseline advanced imaging characteristics (ischemic core volumes, presence of mismatch and HIR) using the Rapid Processing of Perfusion and Diffusion (RAPID) software [15].

## Methods

### Study Cohort

All consecutive stroke patients treated with MT between January 1, 2010 and December 31, 2018 from the local single-center prospective stroke registry were retrospectively assessed for eligibility. STROBE checklist for cohort studies was used to report the present study. Study data are available upon reasonable request to the corresponding authors, and after clearance by the local ethics committee. Inclusion criteria were: (1) acute ischemic stroke treated with MT, (2) available long-term vital status and follow-up time (3) available baseline advanced imaging (including CT perfusion or MRI with diffusion-weighted-imaging (DWI)) and perfusion-imaging and RAPID analysis, (4) available reperfusion status graded by the expanded treatment in cerebral infarction (eTICI). All eligible patients were included in analyses to avoid potential survivorship bias, potentially occurring if only 3‑month survivors are included [16].

### Data Collection

Baseline and 90-day follow-up data were extracted from the local stroke registry. Between September 2019 and May 2020, current vital status was extracted from the National Population Registry, which records the vital status of residents of the country on monthly basis. In addition, two neurologists conducted a telephone interview with surviving patients between September 2019 and June 2020. They obtained long-term functional outcomes using the modified Rankin Scale (mRS) from patients, their next of kin, or health care professionals. Deceased patients at the time of follow-up were assigned a mRS of 6. Long-term favorable functional outcomes were defined as mRS 0–3 and long-term good functional outcomes as mRS 0–2. Health-related quality of life (HRQOL) using the 3‑level version of the EuroQoL 5‑Dimension (EQ-5D-3L) tool was equally assessed [17]. The detailed assessment and interpretation of the HRQOL using EQ-5D-3L in general and in this patient cohort was described somewhere else [18]. The EuroQoL 5‑Dimension (EQ-5D) utility index was derivated from collected data and served to evaluate individual HRQOL [19]. An EQ-5D utility index of 0 indicates deceased patients and an EQ-5D utility index of 1 the best health status possible. A negative EQ-5D utility index value refers to a condition subjectively worse than death [19].

For surviving patients, the follow-up time was defined as the interval between the index ischemic stroke and the last update of the National Population Registry for survival analysis. For outcomes analysis, as the time between the index ischemic stroke and the telephone interview. For deceased patients, the follow-up time was defined as the interval between the index ischemic stroke and the date of death for survival analysis. For outcomes analysis, as the time between the index ischemic stroke and the last update of the National Population Registry.

**Fig. 1:**
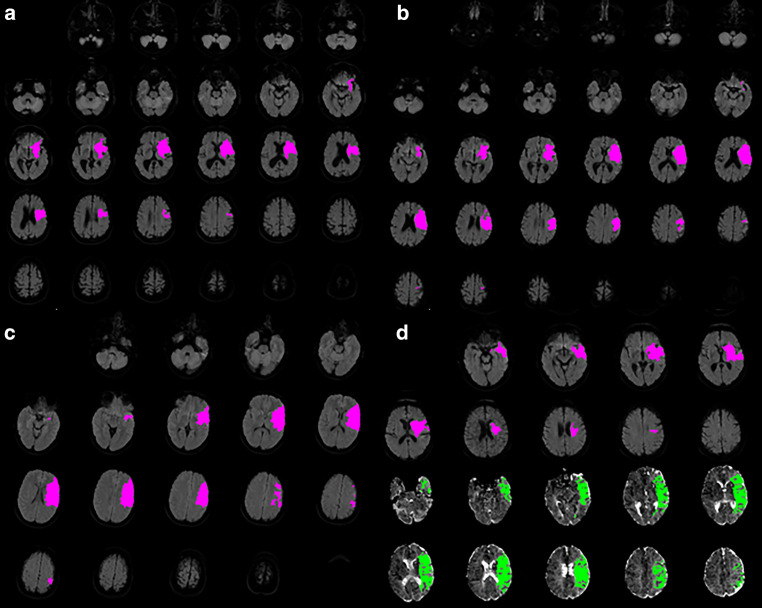
Representation of RAPID cut-offs used to compare the long-term outcome after mechanical thrombectomy according to baseline advanced imaging characteristics. (**a**, **b**, **c**) represent infarct core volumes of approximately 50 ml, 70 ml respectively 100 ml (*pink areas*) assessed here on baseline MRI using diffusion-weighted-imaging. (**d**) represent an absolute mismatch of approximately 50 ml assessed as volume with Tmax > 6 s on perfusion imaging (*green areas*) minus volume with ischemic core volume (*pink areas*)

**Fig. 2:**
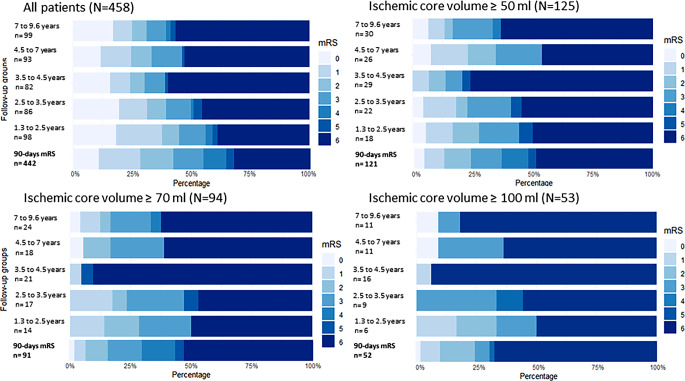
Distribution of short and long-term modified Rankin Scale (*mRS*) stratified by ischemic core volumes on admission. All included patients with available long-term mRS were allocated to 5 follow-up subgroups due to the heterogeneous follow-up time. The mRS-distribution in the long-term follow-up is depicted for all included patients with long-term mRS available, and specifically for those with an ischemic core volume on admission ≥ 50 ml, ≥ 70 ml, and ≥ 100 ml. The short-term mRS from the 90-day control was added at the bottom of each group to appreciate the evolution in the long-term

Initial advanced imaging characteristics were assessed on baseline brain CT and MRI using the RAPID automated post-processing software (iSchemaView, Menlo Park, CA, USA), with predefined cut-offs for both entities. All digital imaging and communications in medicine (DICOMs) of the evaluated patients were transmitted to a single scientific RAPID server. DICOMs were analyzed regarding ischemic core, mismatch volume and HIR at one time. Using CT, the ischemic core volume was determined by relative cerebral blood flow (rCBF) of less than 30% of the corresponding contralateral side [20]. Using MRI, the ischemic core was defined as the volume with apparent diffusion coefficient < 620 × 10^−6^ mm^2^/s on b0/b1000 image [21, 22]. Ischemic core volume cut-offs for analysis were set at 50 ml, 70 ml and 100 ml according to the values generally used in the literature (Fig. 1a, b and c, [1, 2, 23, 24]). Absolute mismatch volume was calculated as volume with T_max_ > 6 s minus volume with a rCBF < 30% (ischemic core) [20]. Relevant mismatch was considered to be present in case of an absolute mismatch volume of > 50 ml (Fig. 1d). The HIR is defined as the ratio between time-to-maximum (T_max_) > 10 s and T_max_ > 6 s in perfusion imaging [25]. A higher percentage of well-perfused tissue and consequently less prolonged blood flow reflect a low HIR and is meant to be associated with robust collaterals [8]. According to previous studies, the dichotomization for low and high HIR was based on the median value of HIR in the present study cohort [8, 25]. After automatic estimation by RAPID, a neuroradiologist manually checked the quality of available perfusion imaging and assessed their plausibility by comparing them with the vessel occlusion site. The expanded treatment in cerebral ischemia (eTICI) score proposed by Liebeskind et al. was core-lab adjudicated to determine the reperfusion grade in the study population [14, 26]. A score of eTICI2b50 or higher was considered to be a successful reperfusion. Stratification by successful reperfusion was used for statistical analyses when the number of patients allowed it. Otherwise, the ordinal reperfusion grade on the eTICI scale was used.

### Statistical Analysis

Baseline characteristics between groups were compared using the chi-square test or Fisher’s exact test when applicable for categorical variables and the Mann-Whitney U test for continuous variables. The characteristics were reported as number and percentage for categorical variables, and median and interquartile range (IQR 25–75%) for continuous variables. Kaplan-Meier curves were plotted to display the mortality rates across the time. Kaplan-Meier estimates were used to estimate mortality rates at 90 days, 1 year and 5 years. The long-term mortality was assessed with survival analysis for the different imaging parameters independently from and stratified by the reperfusion status. To avoid outliers’ bias, patients were right censored at 2556 days (7 years) of follow-up. Adjusted hazard ratios (aHRs) and their 95% confidence intervals (95% CI) were assessed with multivariate Cox regression analysis. For the association between reperfusion status and the long-term functional outcomes, adjusted odds ratios (aOR) and their 95% CI were calculated from multivariate logistic regression. The association between the reperfusion grade and the EQ-5D utility index was determined using the adjusted linear correlation coefficient from multivariate linear regression.

Following clinically relevant covariates were included in all multivariate analysis: age, sex, year of treatment, time between last known well and groin puncture, occlusion site (internal carotid artery, M1-Segment of the middle cerebral artery (MCA), M2-Segment, other anterior occlusion, vertebrobasilar occlusion or other posterior occlusion), low HIR, pre-stroke independence (modified Rankin Scale ≤ 2), national institutes of health stroke scale (NIHSS) score on admission, treatment with intravenous Alteplase, arterial hypertension, dyslipidemia, diabetes, history or active smoking, previous stroke and coronary artery disease. Interaction terms were used to assess the influence of imaging parameters on the association between reperfusion success and long-term mortality. To limit the heterogeneity in the follow-up times of long-term functional outcomes and HRQOL, subgroups with different follow-up times and equal numbers of patients were defined. Analysis involving long-term functional outcomes and EQ-5D utility index were performed using mixed-effects models with defined follow-up time as a random effect.

No imputation was applied to compensate for missing data. Analyses were performed with Stata 16 and R (V. 3.6.0, R Core Team). Statistical significance level was defined as α = 0.05 and all tests were 2‑sided.

## Results

### Study Population

In total, 1303 consecutive patients treated with MT between January 1, 2010 and December 31, 2018 were evaluated (eFigure I—Study flowchart). Twenty-seven patients (2.1%) could not be identified in the National Population Registry, 9 patients (0.7%) deceased with unknown date of death, 731 patients (56.1%) including 395 patients with MRI (54%) and 336 with CT (46%) did not have perfusion imaging on admission (*n* = 307, 42%), RAPID analysis failed or was of poor quality (*n* = 424, 58%), 13 patients (0.9%) had unknown follow-up time and in 4 patients (0.3%) angiographic imaging for the evaluation of the reperfusion grade was missing. The final study cohort included 519 patients (39.8% of evaluated patients) of whom 288 (55.5%) have deceased at the time of the long-term follow-up. Long-term mRS and EQ-5D utility index were available for 88.3% of included patients (*n* = 458, eFigure I). The distribution of patients in the different follow-up groups is represented in Fig. 2. The telephone interview was conducted directly with 89% of surviving patients (*n* = 202) and with their next-of-kin or caregivers in the remaining 11% of patients (*n* = 25).

### Baseline Characteristics

The eTable I (supplementary materials) summarizes the differences between included and excluded patients. There was no difference in mortality rate at 3 months (28.6% versus 25.7%, *P* = 0.29) and in the long-term follow-up (44.5% versus 41%, *P* *=* 0.22). Included patients were older (75.6 years old, IQR 62.8–82.9 versus 73.7, IQR 61.4–81.5), had a shorter time from last known well to admission (120 min, IQR 71–239 versus 169, IQR 87–290), had higher NIHSS on admission (16, IQR 10–20 versus 15, IQR 9–19) and were less likely to have had MRI as baseline imaging (47.6% versus 54.5%). As summarized in Table 1, included patients with successful reperfusion had a shorter time between last known well and admission or groin puncture compared to the included patients with failed reperfusion. Successful reperfusion was more frequent in proximal occlusions and when fewer maneuvers were performed. Based on the median HIR value of included patients (0.43, IQR 0.26–0.58), HIR ≤ 0.43 was defined as low HIR. The distribution of HIR in relation to ischemic core volumes is summarized in eFigure II. Three-month and long-term mortality rate were lower in the group with successful reperfusion (23.6% versus 53.6%, *P* < 0.01; 40.2% versus 66.7% *P* < 0.01, respectively), and they consequently had longer follow-up time (30 months, IQR: 6–58 versus 1 month, IQR: 0.1–25).

**Table 1:** Comparison of baseline characteristics between included patients with failed reperfusion and successful reperfusion

	All (N = 519)	Unsuccessful reperfusion (N = 84)	Successful reperfusion (N = 435)	*P*-value
*Baseline*				
Age at admission (median, IQR)	75.6 (62.8–82.9)	72.2 (61.0–81.0)	75.9 (63.6–83.6)	0.33
Sex (female) (No./total No. (%))	271/519 (52.2)	45/84 (53.6)	226/435 (52.0)	0.81
Independence before stroke (mRS ≤ 2) (No./total No. (%))	452/515 (87.8)	69/82 (84.1)	383/433 (87.6)	0.27
*Risk factors (No/total No. (%))*				
Diabetes	89/518 (17.2)	16/84 (19.0)	73/434 (17.0)	0.64
Hypertension	368/518 (71.0)	57/84 (68.0)	311/434 (71.7)	0.51
Dyslipidemia	287/515 (55.7)	43/83 (51.8)	244/432 (56.5)	0.47
Smoking	120/514 (23.3)	19/82 (23.2)	101/432 (23.4)	1.00
Previous stroke	66/519 (12.7)	10/84 (11.9)	56/435 (12.4)	1.00
CAD	109/516 (21.1)	18/83 (21.7)	9/433 (21.0)	0.88
*Stroke characteristics*				
Time from last known well to admission in min (median, IQR)	120 (71–239)	150 (94–283)	109.5 (69–231)	0.02
NIHSS on admission (median, IQR)	16 (10–20)	15 (9–21)	16 (10–20)	0.44
Time from last known well to groin puncture in min (median, IQR)	218 (161–345)	252.5 (192.5–391.5)	208 (156–335)	0.002
Mechanical thrombectomy beyond 6 h No./total No. (%)	121/511 (23.7)	27/84 (29.8)	94/427 (22)	0.050
MRI as baseline imaging (No./total No. (%))	246/517 (47.6)	37/83 (44.6)	209/434 (48.2)	0.63
Initial ischemic core volume ≥ 50 ml (No./total No. (%))	136/519 (26.2)	25/84 (29.8)	111/435 (25.5)	0.42
Initial ischemic core volume ≥ 70 ml (No./total No. (%))	101/519 (19.5)	19/84 (22.6)	82/435 (18.9)	0.45
Initial ischemic core volume ≥ 100 ml (No./total No. (%))	60/519 (11.56)	14/84 (16.7)	46/435 (10.6)	0.13
HIR (median, IQR)	0.43 (0.26–0.58)	0.40 (0.26–0.59)	0.44 (0.26–0.57)	0.99
Low HIR (≤ 0.43) (No./total No. (%))	259/519 (49.9)	46/84 (54.8)	213/435 (49)	0.34
Relevant mismatch (≥ 50 ml) (No./total No. (%))	82/136 (60.29)	12/25 (48.0)	70/111 (63.1)	0.18
*Site of occlusion (No./total No. (%))*				
ICA	132/519 (25.4)	25/84 (29.8)	107/435 (24.6)	< 0.001
M1	263/519 (50.7)	28/84 (33.3)	235/435 (54.0)	
M2	83/519 (16.0)	23/84 (27.4)	60/435 (13.8)	
Other anterior occlusion	7/519 (1.3)	3/84 (3.6)	4/435 (0.9)	
Vertebrobasilar occlusion	23/519 (4.4)	1/84 (1.2)	22/435 (5.1)	
Other posterior occlusion	11/519 (2.1)	4/84 (4.8)	7/435 (1.6)	
*Stroke treatment*				
IVT-bridging (No./total No. (%))	206 (39.7)	25/84 (29.8)	181/435 (41.6)	0.051
Number of maneuvers (median, IQR)	1 (1–2)	2 (1–4)	1 (1–2)	< 0.001
*Long-term mortality*				
3‑months mortality (No./total No. (%))	143/518 (27.6)	45/84 (53.6)	98/434 (22.6)	< 0.001
Long-term follow-up time in months (median, IQR)	28 (1–55)	1 (0.1–25)	30 (6–58)	< 0.001
Long-term mortality (No./total No. (%))	231/519 (44.5)	56/84 (66.7)	175/435 (40.2)	< 0.001

*CAD* coronary artery disease; *ICA* internal carotid artery; *HIR* hypoperfusion intensity ratio; *IQR* interquartile range; *IVT* intravenous thrombolysis; *mRS* modified Rankin Scale; *M1 and M2* first and second segment of the middle cerebral artery; *NIHSS* National Institutes of Health Stroke Scale

### Long-term Mortality According to Ischemic Core Volumes

**Fig. 3:**
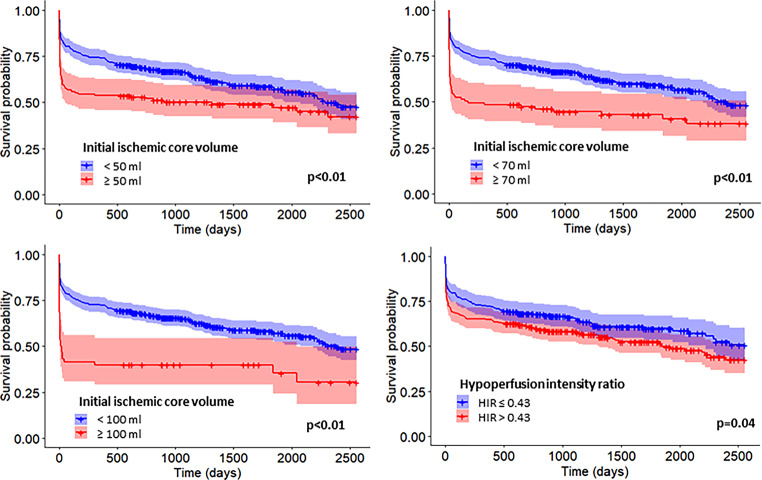
Survival curves for long-term mortality according to the different initial ischemic core volumes and the hypoperfusion intensity ratio. Survival curves (with 95% CI) with strata for the different ischemic core volumes cut-offs (50 ml, 70 ml and 100 ml) and collaterals status defined by the hypoperfusion intensity ratio (*HIR*) with HIR ≤ 0.43 indicating good collaterals and > 0.43 indicating poor collaterals. A larger initial ischemic core volume was associated with higher mortality in the long-term: **a** < 50 ml versus ≥ 50 ml (log-rank test, P = 0.01); **b** < 70 ml versus ≥ 70 ml (log-rank test, P < 0.01); **c** < 100 ml versus ≥ 100 ml (log-rank test, P < 0.01). An influence of the collaterals status on long-term mortality was also observed (**d** log-rank test, P = 0.04)

Independently of the reperfusion status, the mortality rate at three months, one year and five years follow-up time differed depending on initial ischemic core volume as summarized in eFigure III. The mortality rate over the follow-up time was higher in patients with larger ischemic core volumes on admission (Fig. 3a, b and c, log-rank tests *P* *<* 0.01). The multivariate Cox regression analysis with adjustment for prespecified covariates demonstrated an association between successful reperfusion and lower long-term mortality rate for all ischemic core volumes assessed (eFigure IV). For ischemic core volume ≥ 50 ml the aHR was 0.29 (95% CI 0.16–0.53, eFigure IV-A), for ≥ 70 ml aHR 0.20 (95% CI 0.10–0.44, eFigure IV-B) and for ≥ 100 ml aHR 0.26 (95% CI 0.08–0.87, eFigure IV-C). There was no interaction of the initial ischemic core volumes on the association between the reperfusion status and the long-term mortality (≥ 50 ml, *P* *=* 0.11; ≥ 70 ml, *P* *=* 0.27; ≥ 100 ml, *P* *=* 0.55). Higher age at admission and previous stroke were associated with long-term mortality in all analyses (eFigure IV). The number of patients who were treated beyond 6 h and had an ischemic core volume larger than the predefined cut-offs was too small to perform subgroups analyses (≥ 50 ml, *n* = 28; ≥ 70 ml, *n* = 21; ≥ 100 ml, *n* = 10).

### Long-term Functional Outcomes According to Ischemic Core Volumes

Of the 458 patients with available long-term mRS, 212 (46.3%) had a long-term favorable functional outcome. Favorable outcomes were reported in 37.6% of ischemic core volume ≥ 50 ml (*n* = 47/125), in 33% of volume ≥ 70 ml (*n* = 31/94) and in 24.5% of volume ≥ 100 ml (*n* = 13/53). Figure 2 depicted the distribution of mRS at 90-days and in the different long-term follow-up subgroups for all patients and for large ischemic core volumes only (≥ 50 ml, ≥ 70 ml and ≥ 100 ml). Differences in 90-days functional outcomes and long-term functional outcomes according to ischemic core volumes are summarized in eTable II. In the multivariate logistic regression increasing reperfusion grade was associated with favorable outcomes in patients with ischemic core volume ≥ 50 ml (aOR 2.54, 95% CI 1.47–4.38) and ≥ 70 ml (aOR 3.58, 95% CI 1.64–7.83; eFigure V). Increasing reperfusion grade were also associated with good outcomes in ischemic core volume ≥ 50 ml (aOR 3.58, 95% CI 1.64–7.83) and ≥ 70 ml (aOR 2.98, 95% CI 1.19–7.44; eFigure V). Interaction analyses did not identify an influence of the initial ischemic core volume on the relation between final eTICI grade and long-term functional outcomes (eFigure V). The number of patients in the group with infarcts ≥ 100 mL was insufficient to enable multivariate analysis.

### Long-term Health-related Quality of Life According to Ischemic Core Volumes

The multivariate linear regression analysis demonstrated an association between higher reperfusion grade and higher EQ-5D utility index in patients with ischemic core volumes ≥ 50 ml, ≥ 70 ml and ≥ 100 ml (adjusted linear correlation coefficient 0.07, 95% CI 0.04–0.10; 0.09, 95% CI 0.06–0.13 and 0.06, 95% CI 0.02–0.10, respectively). No interaction of the ischemic core volumes was found in the association between the EQ-5D utility index and the eTICI grade (*P* Interaction for ≥ 50 ml = 0.99, ≥ 70 ml = 0.83 and ≥ 100 ml = 0.43).

### Long-term Outcomes and Hypoperfusion Intensity Ratio

Independently of the ischemic core volume and reperfusion status, the long-term mortality was lower in the case of low HIR (Fig. 3d, log-rank test *P* *=* 0.04). Low HIR was not associated with a lower long-term mortality rate in the adjusted analysis (eFigure IV). Nevertheless, a direct interaction of the ischemic core volumes on the association between the collateral status and long-term mortality was present for all volumes larger than the predefined cut-offs (≥ 50 ml, *P* *<* 0.01; ≥ 70 ml, *P* *<* 0.01; ≥ 100 ml, *P* *<* 0.01). Furthermore, low HIR was neither associated with long-term favorable outcomes (aOR 0.91, 95% CI 0.54–1.53) nor with long-term good outcomes (aOR 0.92, 95% CI 0.54–1.55). No association was found between low HIR and EQ-5D utility index (adjusted linear correlation coefficient 0.01. 95% CI −0.05–0.81).

### Long-term Outcomes and Relevant Mismatch

The effect of successful reperfusion on the long-term mortality was demonstrated exclusively in presence of relevant mismatch (eFigure VI, log-rank test *P* *<* 0.01) and was associated with an aHR of 0.17 (95% CI 0.07–0.44, eFigure VII). Multivariate analyses regarding the effect of reperfusion on long-term functional outcomes in case of mismatch were not possible because of the small number of patients in the group without relevant mismatch. The effect of reperfusion on long-term HRQOL was more significant in the presence than in the absence of relevant mismatch (adjusted linear correlation coefficient 0.10, 95% CI 0.06–0.15 versus 0.05, 95% CI 0.01–0.10, respectively).

## Discussion

The main findings of this study are: (1) The positive long-term effects of higher reperfusion grade are also present in large ischemic core volumes; (2) The relevance of mismatch status in the selection of patients for MT is also visible in the long-term (3) A favorable collateral status (defined by a low HIR) seems to be associated with lower long-term mortality.

Regarding the effectiveness of MT in large infarcts, some studies (including the recently published ANGEL-ASPECT trial) reported the benefit in ischemic core volumes < 100 ml and hypothesized a potential benefit of MT in volumes up to 150 ml.[23, 27, 28]. Our study, based on advanced imaging characteristics, described an association between higher reperfusion grade and better long-term outcomes (lower mortality, better functional outcomes and better HRQOL). The associations were not only present in relatively smaller ischemic core volumes but also in ischemic core volumes ≥ 100 ml when the analyses were possible. As the indication for treatment of large ischemic infarcts (particularly if ≥ 70 ml) remains unclear, our results demonstrated a long-term benefit in these patients. Furthermore, as recently shown by the SATIN (Stroke treatment Assessments prior to Thrombectomy In Neurointervention) study, neurointerventionalists tend to predict 90-day outcomes in MT-patients negatively [29]. Our findings in the long-term could consequently reassure neurointerventionalits to perform thrombectomy in cases of large ischemic core at baseline and thus bring the benefits of MT to more stroke patients with an inaccurately assumed poor prognosis. The consideration of advanced imaging in patients treated with MT within 6 h after last known well is currently not included in the AHA recommendations. Despite this, it has enabled the extension of the MT indication beyond 6 h and remains a relevant tool to describe important imaging characteristics, such as the tissue at risk in borderline cases [1, 2, 12, 30, 31]. Randomized controlled trials (RCT), like DEFUSE 3 (Endovascular Therapy Following Imaging Evaluation for Ischemic Stroke 3) and DAWN (Clinical Mismatch in the Triage of Wake Up and Late Presenting Strokes Undergoing Neurointervention With Trevo), which extended the time window for MT up to 16 h and 24 h, respectively, included patients with relatively small ischemic core (< 1/3 of MCA territory or < 70 ml) and the presence of a relevant mismatch [32]. Consequently, the indication for thrombectomy in large infarcts, especially in the late time window, remains unclear, even if growing evidence is surging [18]. Most recently, three RCTs (ANGEL ASPECT, SELECT2) and RESCUE-Japan LIMIT demonstrated the benefit of MT at 90 days in patients with large infarcts defined by an Alberta Stroke Program Early Computed Tomographic Score (ASPECTS) of 3 to 5 [28, 33, 34]. Nevertheless, even though the ASPECTS is frequently used in clinical trials, it remains a coarse estimate of the ischemic core volume [35]. Compared to assigning ASPECTS on non-contrast CT, CT perfusion allows accurate ischemic core volume estimation and was associated with better prediction of the final infarct volume and complications such as symptomatic hemorrhage, neurologic deterioration and mortality at 90 days [23, 36]. Further studies showed that compared to CT perfusion, MRI imaging using DWI is associated with better prediction of good outcomes and can better delineate ischemic core [23, 37]. In our study, MRI with DWI and MR-perfusion was performed in approximately half of the patients at baseline. Thus, the large number of MRI scans performed in our study could have increased the quality of the presented results.

A relevant mismatch was associated with a reduction in long-term mortality and better HRQOL in case of higher reperfusion grade in our study. This association is in line with the DEFUSE 2 (Endovascular Therapy Following Imaging Evaluation for Ischemic Stroke 2) study, which demonstrated a better short-term functional outcome when a significant mismatch before MT was present [38].

In our study, low HIR predicted lower long-term mortality (Fig. 3d). However, contrary to current evidence, a low HIR was not associated with better outcomes after adjustment for reperfusion status and other covariates [9, 39]. As summarized in eFigure II, patients with low HIR in subgroups with larger ischemic core volumes were underrepresented compared to patients with high HIR. The rationale for this underrepresentation might be that patients with a later presentation who are still eligible for MT are those with smaller ischemic core, relatively preserved penumbra and lower symptoms severity, as hypothesized by Monteiro et al. [40].

### Limitations

Firstly, this was a monocentric and retrospective study with a small sample size due to the restricted patient population of interest. Due to the small number of patients included, the power of the study is reduced and the observational nature of the study makes the results susceptible to hidden bias. Secondly, non-conducted perfusion imaging, motion artifacts during acquisition and software errors in post-processing of imaging (algorithm threshold for displaying core infarct and poor contrast bolus) led to the exclusion of a consequent number of patients, limiting the use of the study results in the clinical routine [41]. Thirdly, the small number of patients did not allow subgroup analysis of patients treated beyond 6 h, who represents a subgroup of particular interest currently in the field of stroke research. Finally, the inclusion of MT patients only led to an underrepresentation of patients with low HIR in the subgroup with large ischemic cores. The interpretation of the results related to HIR should consequently be made with precaution. Further studies investigating advanced imaging-based long-term outcomes after MT and including data acquired after the extension of the MT time window beyond 6 h are warranted.

## Conclusion

In line with the studies reporting short-term benefits, our study demonstrated an association between successful reperfusion in stroke patients with large ischemic core volumes and better outcome. This association was stable in patients with a relevant mismatch, but further data is needed for patient without such a mismatch. Our analyses do not show large heterogeneity of the effect size of successful reperfusion on long-term outcomes and hence do not support patient deselection based on large core alone.

### Supplementary Information

eFigures I–VII and eTables I–II

STROBE Checklist

## Abbreviations

EQ-5D: EuroQoL 5‑Dimension
eTICI: Expanded Treatment In Cerebral Infarction
HIR: Hypoperfusion Intensity Ratio
HRQOL: Health-related quality of life
MT: Mechanical Thrombectomy
mRS: Modified Rankin Scale
NIHSS: National Institutes of Health Stroke Scale
RAPID: Rapid Processing of Perfusion and Diffusion
RCT: Randomized Controlled Trial
