# Supplementary material for: Long-Term Effect of Mechanical Thrombectomy in Stroke Patients According to Advanced Imaging Characteristics
Source: Clin Neuroradiol. 2023 Aug 29;34(1):105–14. doi: 10.1007/s00062-023-01337-4 (PMC10881753; doi:10.1007/s00062-023-01337-4)
Supplement: Supplementary file 1 — eFigures I–VII and eTables I–II [file 62_2023_1337_MOESM1_ESM.docx]

**SUPPLEMENTAL MATERIALS**

**Long-Term Effect of Mechanical Thrombectomy in Stroke Patients according to Advanced Imaging Characteristics.**

**Supplementary Figures**


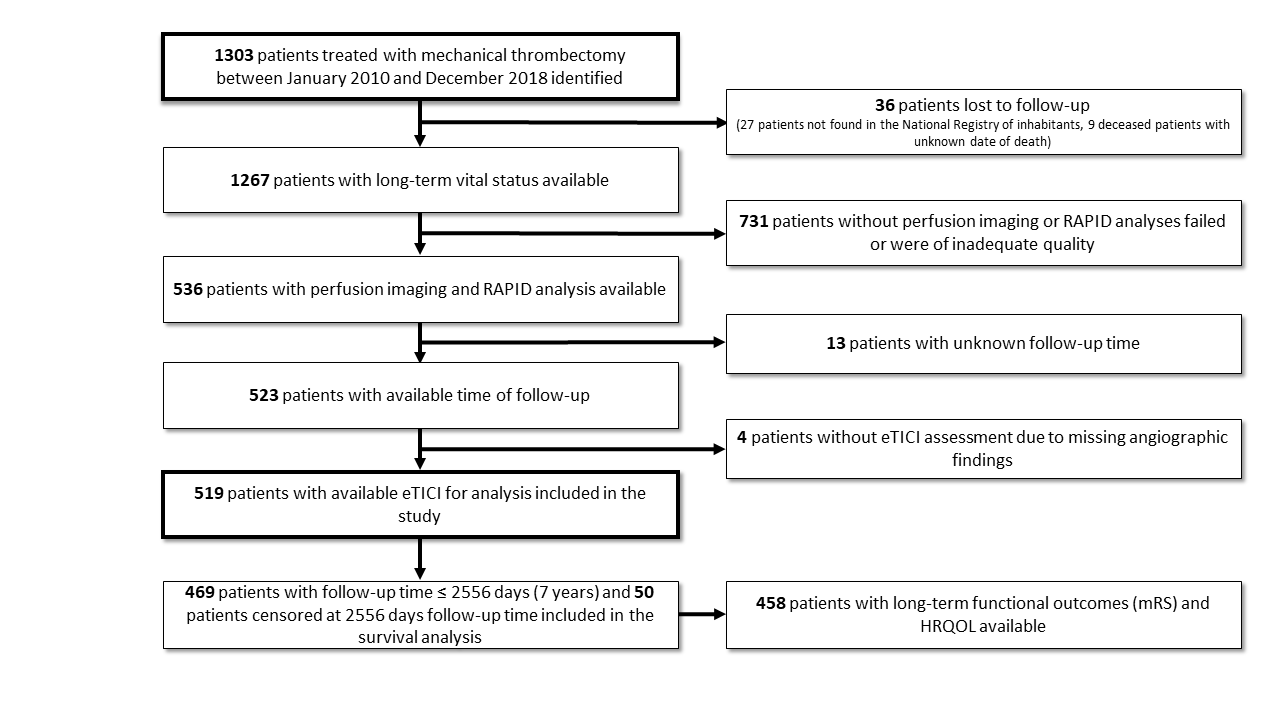


**eFigure I – Study flowchart**Process of inclusion and exclusion in the study. eTICI indicates expanded Treatment in Cerebral Infarction; HRQOL, health-related quality of life; mRS, modified Rankin Scale; and RAPID, rapid processing of perfusion and diffusion software.

**
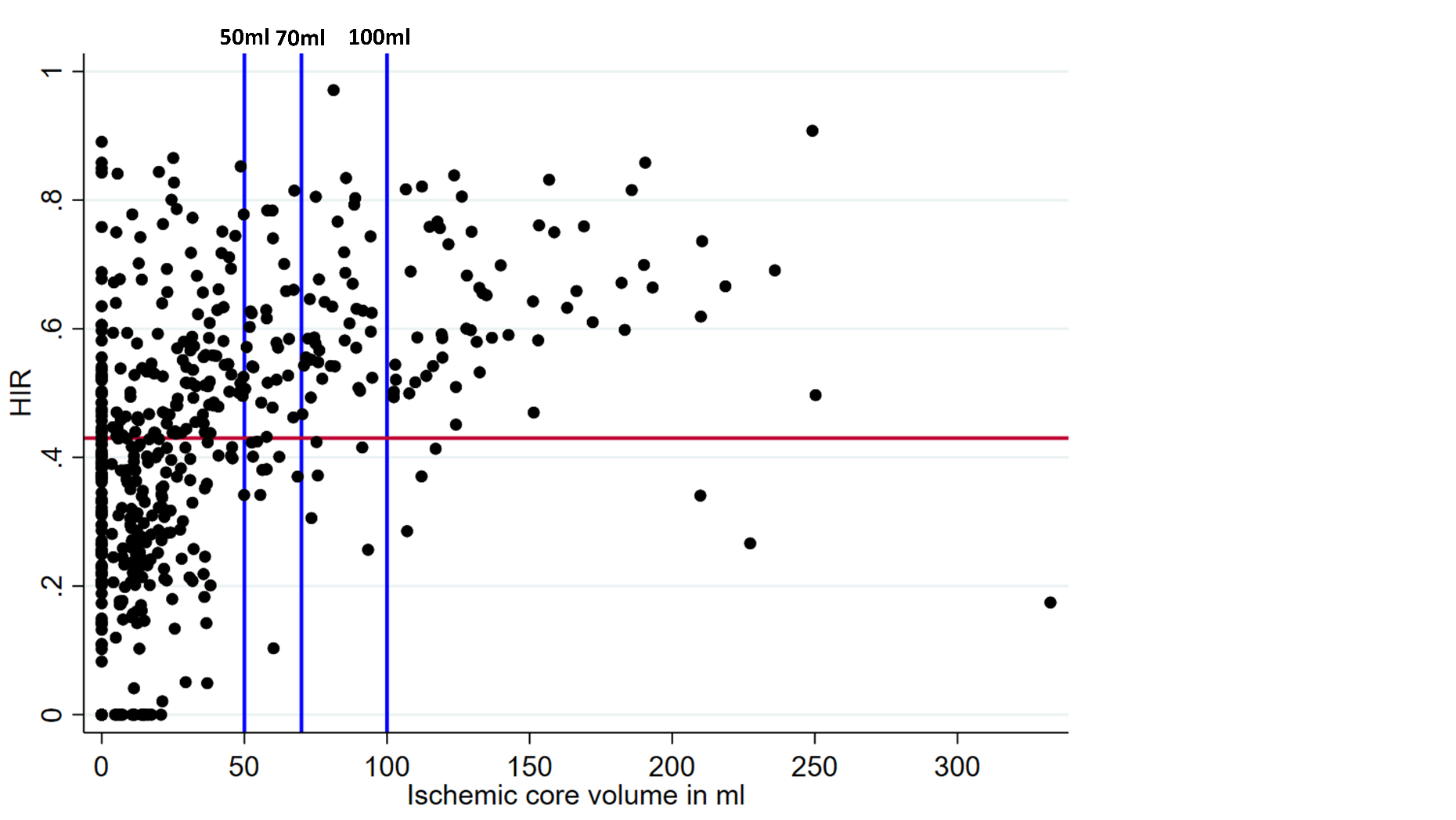
**

**eFigure II – Distribution of hypoperfusion intensity ratio in relation to the initial ischemic core volumes**The blue lines represent the different volume cut-offs specific to the study (50ml, 70ml and 100ml). The red line represents the cut-off for the hypoperfusion intensity ratio (HIR) specific to the study population, set at 0.43. Patients with a large ischemic core volume and low HIR were generally not candidates for thrombectomy because of the delayed arrival at hospital and are therefore underrepresented in the study population.


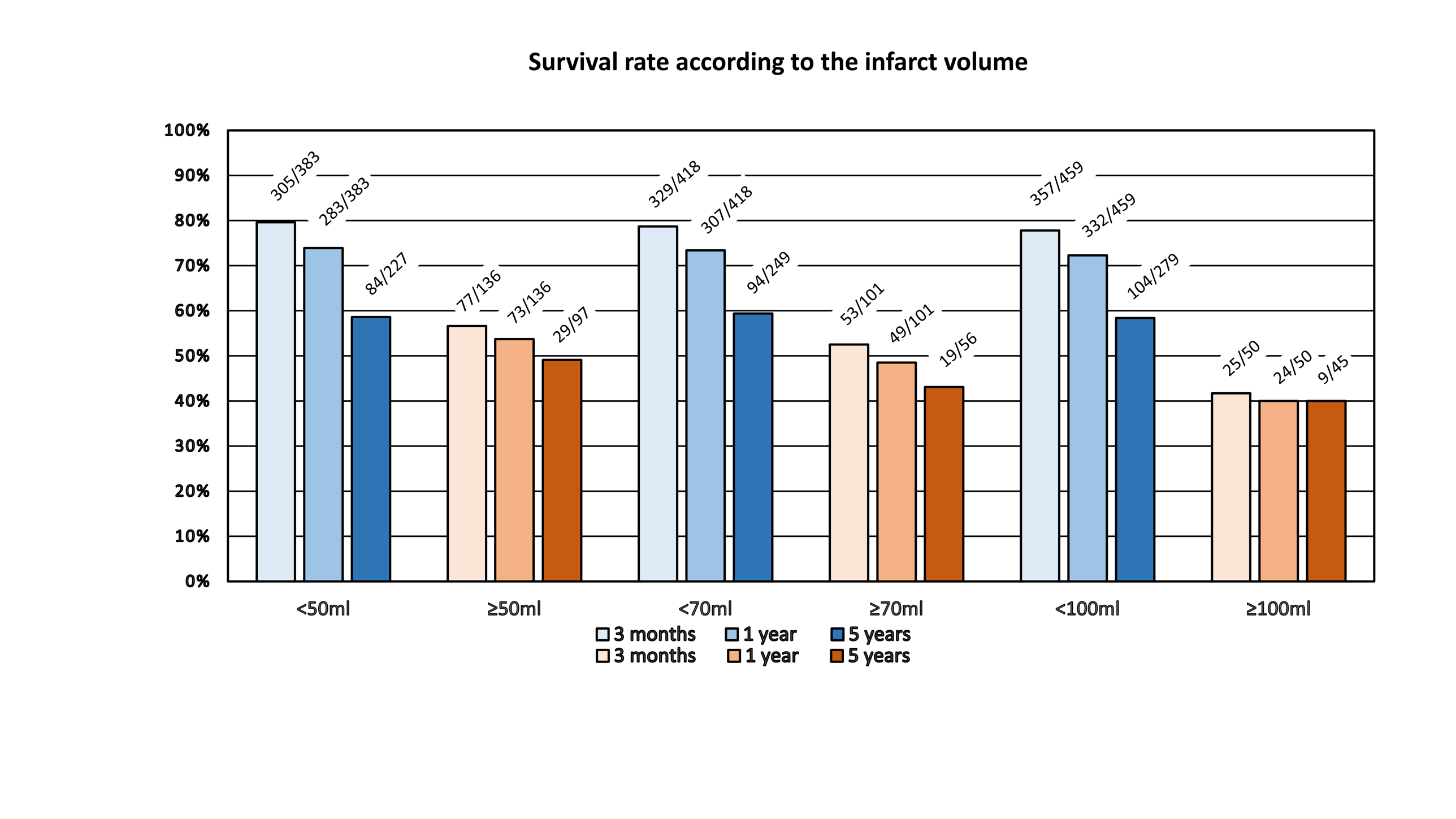


**eFigure III –** **Survival rates for the different ischemic core volumes across the follow-up time**The survival rates are summarized for the following follow-up times: three months, one year and five years. Ischemic core volumes below the cut-offs defined at 50ml, 70ml and 100ml are shown in blue and ischemic core volumes above the cut-offs in orange. The number of survivors per group and follow-up time is reported above the corresponding bar.


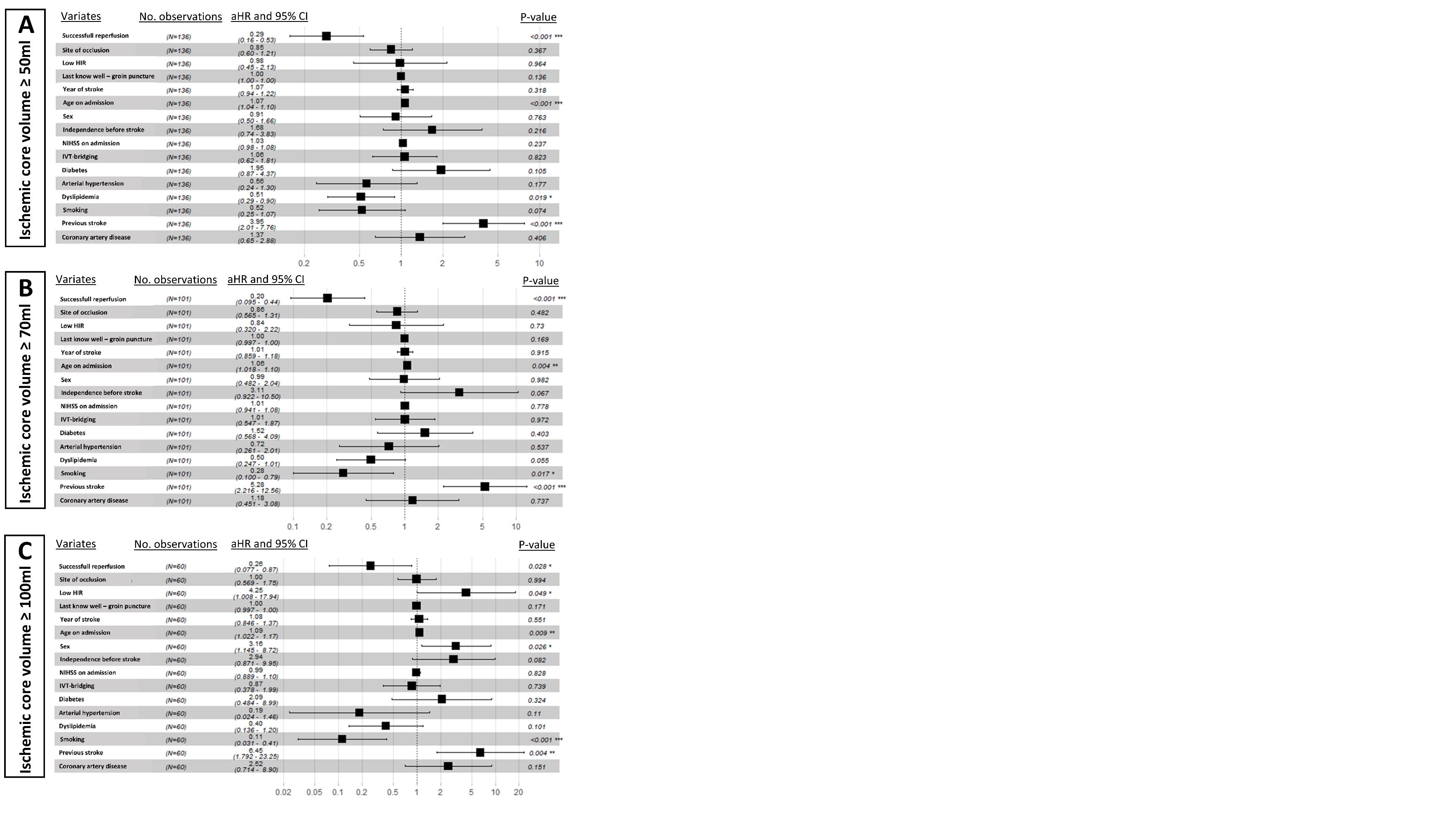


**eFigure IV** **– Association between successful reperfusion and long-term mortality according to different initial ischemic core volumes**

Adjusted hazard ratios (aHR) from the multivariate Cox regression analyses between successful reperfusion (assessed by eTICI ≥ 2b50) and long-term mortality depending on the initial ischemic core volume ≥ 50ml (A), ≥ 70ml (B) and ≥ 100ml (C). Analyses were adjusted for the site of occlusion, low HIR (≤0.43), time from last known well to groin puncture, year of stroke, age on admission, sex, independence before stroke (defined as mRS 0-2), NIHSS on admission, bridging therapy with IVT, diabetes, arterial hypertension, dyslipidemia, smoking, previous stroke and coronary artery disease. eTICI score indicates expanded Treatment in Cerebral Infarction score; HIR, hypoperfusion intensity ratio; IVT, intravenous thrombolysis; mRS, modified Rankin Scale; NIHSS, national institutes of health stroke scale; No. observations, number of observations; 95% CI, 95% confidence intervals.


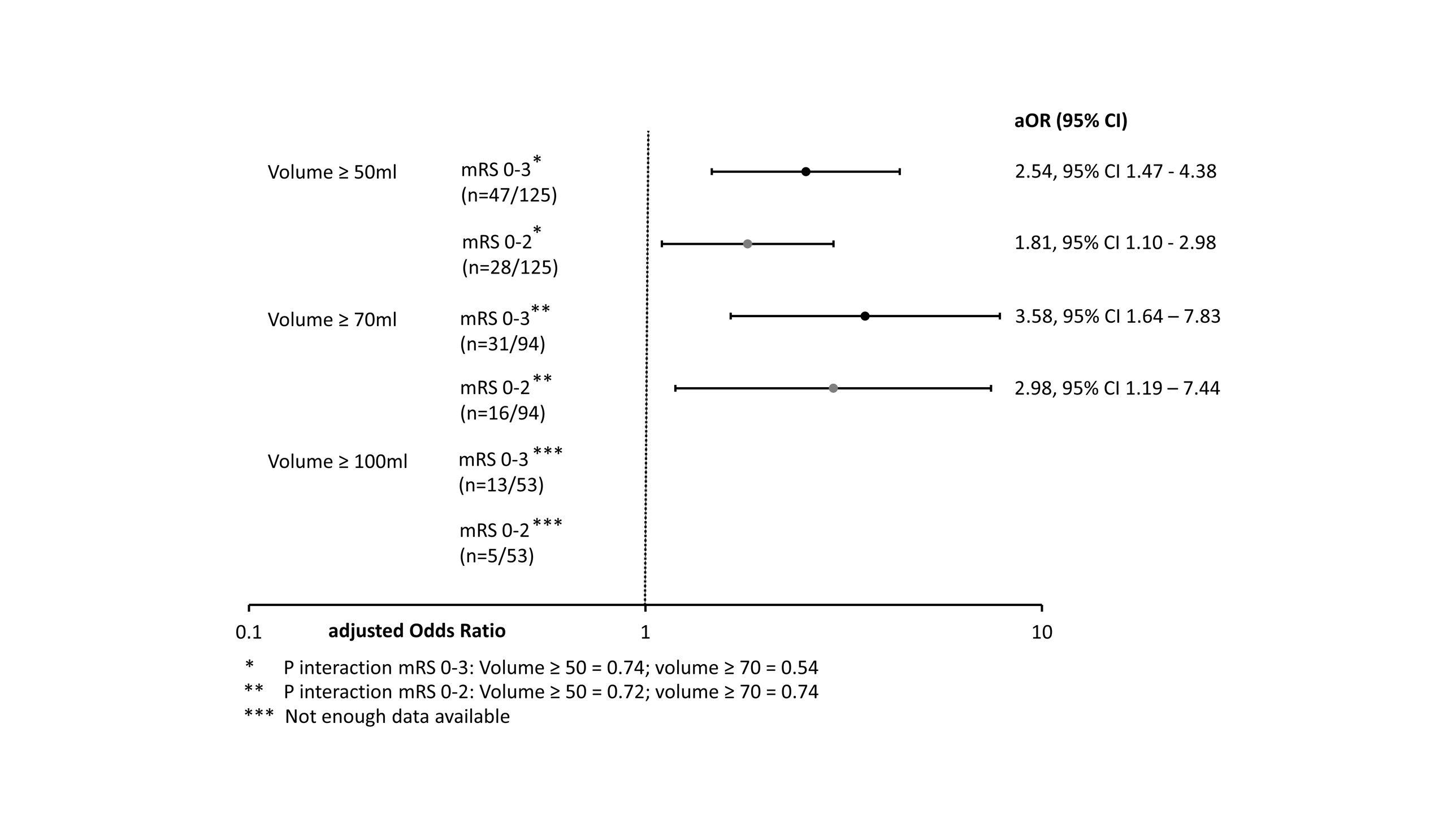


**eFigure V–** **Correlation between eTICI grade and functional outcome stratified by ischemic core volume at admission**
Adjusted odds ratios (aOR) between final reperfusion grade (assessed by eTICI) and favorable functional outcome (defined as mRS 0–3) or good functional outcome (defined as mRS 0-2) for patients with ischemic core volume ≥ 50ml, ≥ 70ml and ≥ 100ml at admission. All follow-up groups were assessed in the mixed-effects model to compensate for the small number of patients in each follow-up group. Not enough patients with long-term mRS were available in the group with infarcts ≥100 mL to perform multivariate analyses. Higher reperfusion grade was associated with favorable and good long-term outcomes in patients with ischemic core volume ≥50ml and ≥ 70ml. Interaction analyses (* and **) did not identify a substantial influence of the initial ischemic core volume on the relation between final eTICI and functional outcomes. aOR indicates adjusted Odds Ratio; eTICI, expanded Treatment in Cerebral Infarction; mRS, modified Rankin Scale; *P interaction, P-value for interaction; 95% CI, 95% confidence intervals.


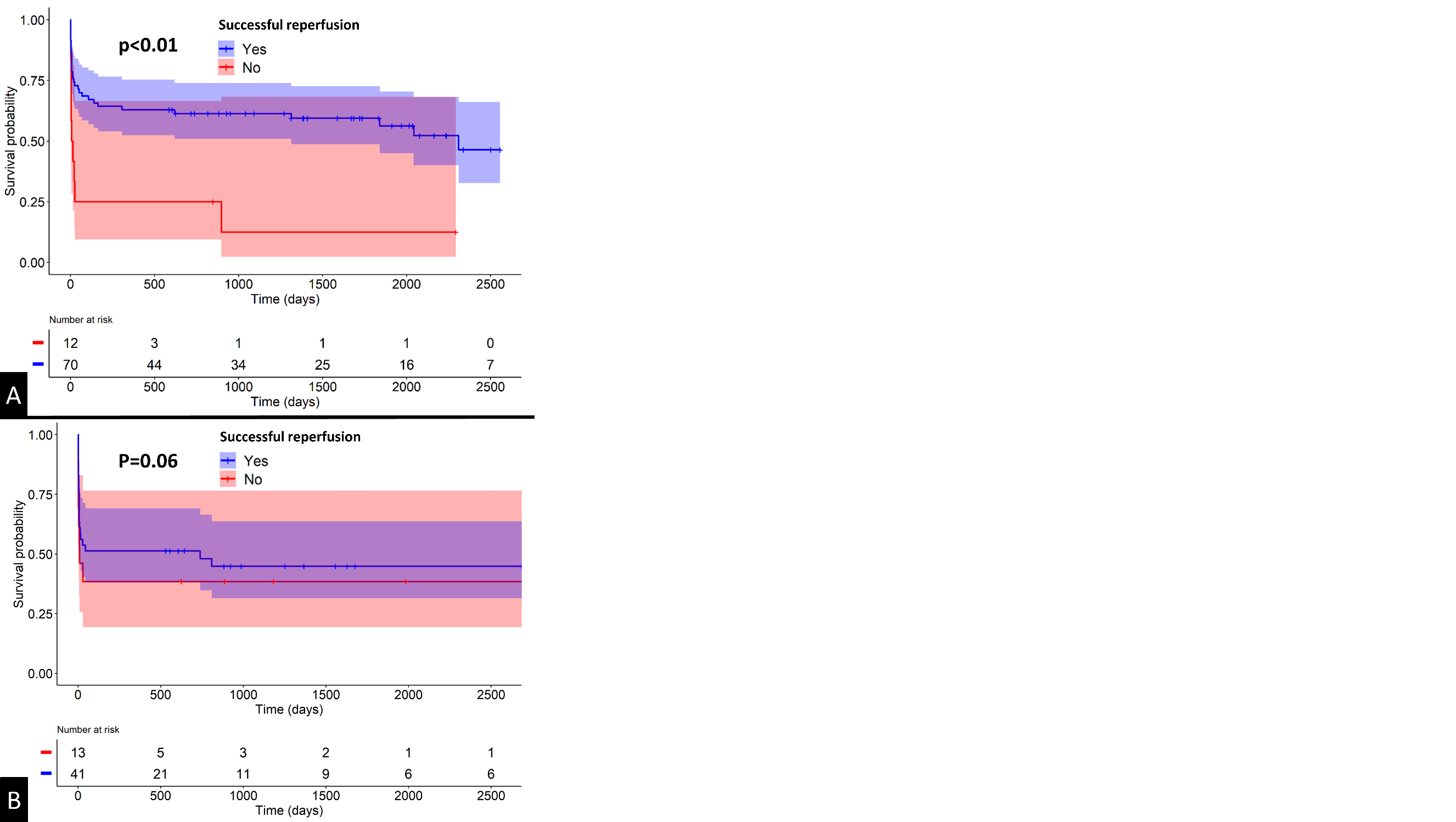


**eFigure VI – Survival curves for long-term mortality in patients with versus without relevant mismatch stratified by reperfusion success**Survival curves (with 95% CI) for patients with and without relevant mismatch (Figure 4A respectively Figure 4B) with strata for successful reperfusion. Relevant mismatch was defined as an absolute mismatch > 50ml. Successful reperfusion influenced the long-term mortality in patients with relevant mismatch status (log-rank test, *P<*0.01) but not in those without relevant mismatch (log-rank test, *P=*0.06).


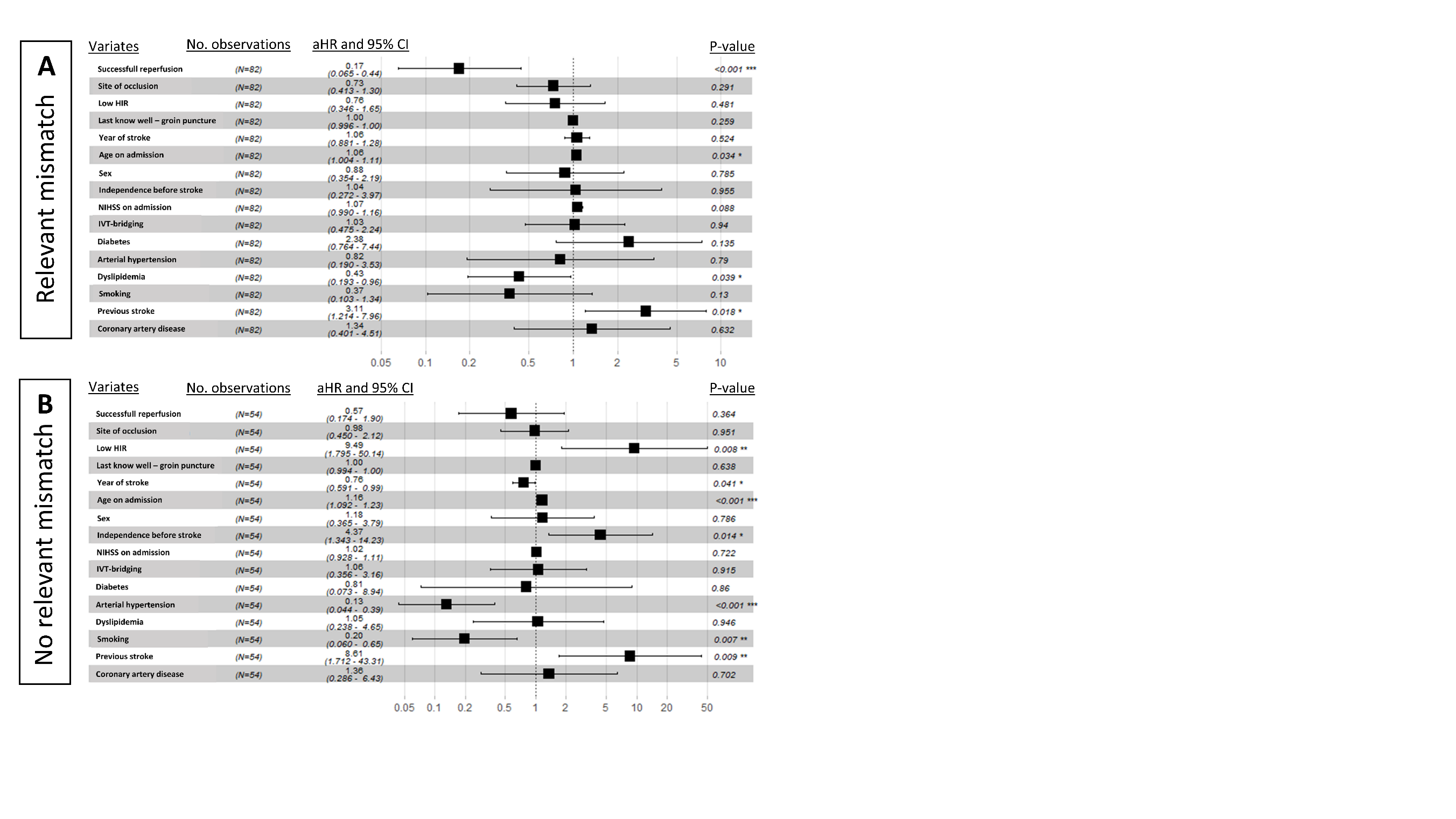


**eFigure VII – Correlation between successful reperfusion and long-term mortality in the presence and absence of relevant mismatch**
Adjusted hazard ratios (aHR) from the multivariate Cox regression analyses between successful reperfusion (assessed by eTICI ≥ 2b50) and long-term mortality in presence of relevant mismatch (A) and in absence of relevant mismatch (B) on baseline imaging. Analyses were adjusted for the site of occlusion, low HIR (≤0.43), time from last known well to groin puncture, year of stroke, age on admission, sex, independence before stroke (defined as mRS 0-2), NIHSS on admission, bridging therapy with IVT, diabetes, arterial hypertension, dyslipidemia, smoking, previous stroke and coronary artery disease. eTICI score indicates expanded Treatment in Cerebral Infarction score; HIR, hypoperfusion intensity ratio; IVT, intravenous thrombolysis; mRS, modified Rankin Scale; NIHSS, national institutes of health stroke scale; No. observations, number of observations; 95% CI, 95% confidence intervals.

**Supplementary Tables**

**eTable I** – Comparison of baseline characteristics between excluded and included patients.

|  | All (N = 1303) | Excluded patients  (N = 784) | Included patients  (N = 519) | *P*-value |
| --- | --- | --- | --- | --- |
| Baseline |  |  |  |  |
| Age at admission (median, IQR) | 74.21 (61.97-82.06) | 73.69 (61.36-81.52) | 75.6 (62.8-82.9) | 0.028 |
| Sex (female) No. / total No. (%) | 648/1303 (49.7%) | 377/784 (48.1%) | 271/519 (52.2%) | 0.16 |
| Independence before stroke (mRS≤2)  No. / total No. (%) | 1149/1297 (88.59%) | 697/782 (89.1%) | 452/515 (87.8%) | 0.48 |
| Risk factors  No / total No. (%) |  |  |  |  |
| Diabetes | 225/1299 (17.3%) | 136/781 (17.4%) | 89/518 (17.2%) | 0.94 |
| Hypertension | 909/1300 (69.9%) | 541/782 (69.2%) | 368/518 (71.0%) | 0.50 |
| Dyslipidemia | 722/1292 (55.9%) | 435/777 (56.0%) | 287/515 (55.7%) | 0.95 |
| Smoking | 320/1293 (24.8%) | 200/779 (25.7%) | 120/514 (23.3%) | 0.36 |
| Previous stroke | 161/1300 (12.4%) | 95/781 (12.2%) | 66/519 (12.7%) | 0.80 |
| CAD | 246/1292 (19%) | 137/776 (17.7%) | 109/516 (21.1%) | 0.13 |
| Stroke characteristics |  |  |  |  |
| Time from last known well to admission in min (median, IQR) | 150.5 (79-270) | 169 (87-290) | 120 (71-239) | <0.001 |
| NIHSS on admission (median, IQR) | 15 (9-20) | 15 (9-19) | 16 (10-20) | 0.017 |
| Time from last known well to groin puncture in min (median, IQR) | 238 (173-364) | 250 (180-382) | 218 (161-345) | <0.001 |
| Mechanical thrombectomybeyond 6 hours  No. / total No. (%) | 334/1277 (26.2%) | 213/766 (27.8%) | 121/511 (23.7%) | 0.10 |
| MRI as baseline imaging No. / total No. (%) | 672/1299 (51.7%) | 426/782 (54.5%) | 246/517 (47.6%) | 0.017 |
| **Site of occlusion**  No. / total No. (%) | | | | |
| ICA | 315/1303 (24.2%) | 183/784 (23.3%) | 132/519 (25.4%) | 0.16 |
| M1 | 671/1303 (51.5%) | 408/784 (52.0%) | 263/519 (50.7%) |  |
| M2 | 199/1303 (15.3%) | 116/784 (14.8%) | 83/519 (16.0%) |  |
| Other anterior occlusion | 12/1303 (0.9%) | 5/784 (0.6%) | 7/519 (1.3%) |  |
| Vertebrobasilar occlusion | 82/1303 (6.3%) | 59/784 (7.5%) | 23/519 (4.4%) |  |
| Other posterior occlusion | 24/1303 (1.8%) | 13/784 (1.7%) | 11/519 (2.1%) |  |
| Stroke treatment |  |  |  |  |
| IVT-bridging  No. / total No. (%) | 520/1303 (39.9%) | 314/784 (40.1%) | 206/519 (39.7%) | 0.91 |
| Number of maneuvers  (median, IQR) | 1 (1-2) | 1 (1-2) | 1 (1-2) | 0.12 |
| Long-term mortality | | | | |
| 3-months mortality  No. / total No. (%) | 339/1294 (26.2%) | 196/776 (25.3%) | 143/518 (27.6%) | 0.37 |
| Long-term follow-up time in months (median, IQR) | 30 (3-51) | 32 (3-47) | 28 (1-55) | 0.31 |
| Long-term mortality No. / total No. (%) | 541/1276 (57.6%) | 310/757 (41%) | 231/519 (44.5%) | 0.23 |
| CAD indicates coronary artery disease; ICA, internal carotid artery; IQR, interquartile range; IVT, intravenous thrombolysis; mRS, modified Rankin Scale M1 and M2, first and second segment of the middle cerebral artery; NIHSS, National Institutes of Health Stroke Scale | | | | |

**eTable II** - Comparison of short and long-term outcomes between different ischemic core volumes at admission.

|  | All (N = 458) | Ischemic core volume < 50ml  (N = 333) | Ischemic core volume ≥ 50ml  (N = 125) | *P*-value | Ischemic core volume < 70ml  (N = 364) | Ischemic core volume ≥ 70ml  (N = 94) | *P*-value | Ischemic core volume < 100ml  (N = 405) | Ischemic core volume ≥ 100ml  (N = 53) | *P*-value |
| --- | --- | --- | --- | --- | --- | --- | --- | --- | --- | --- |
| Short-term outcomes of mechanical thrombectomy | | | | | | | | | | |
| Successful reperfusion  No. / total No. (%) | 380/458 (83%) | 278/333 (83.5%) | 102/125 (81.6%) | 0.68 | 304/364 (83.5%) | 76/94  (80.9%) | 0.54 | 340/405  (84%) | 40/53  (75.5%) | 0.12 |
| eTICI (median, IQR) | 4 (3-6) | 4 (3-6) | 4 (3-5) | 0.65 | 4 (3-6) | 4 (3-6) | 0.74 | 4 (3-6) | 4 (3-5) | 0.46 |
| 90-days mRS (median, IQR) | 3 (1-6) | 3 (1-6) | 6 (3-6) | <0.001 | 3 (1-6) | 6 (3-6) | <0.001 | 3 (1-6) | 6 (4-6) | <0.001 |
| Long-term functional outcomes | | | | | | | | | | |
| Long-term mRS (median, IQR) | 6 (1-6) | 4 (1–6) | 6 (3–6) | <0.001 | 4 (1-6) | 6 (3-6) | <0.001 | 4 (1-6) | 6 (5-6) | <0.001 |
| Good functional outcome (mRS 0–2) No. / total No. (%) | 165/458 (36%) | 137/333 (49.5%) | 28/125 (22.4%) | <0.001 | 149/364 (40.9%) | 16/94 (17%) | <0.001 | 160/405  (39.5%) | 5/53  (9.4%) | <0.001 |
| Favorable functional outcome (mRS 0–3) No. / total No. (%) | 212/458 (46.3%) | 165/333 (24.4%) | 47/125 (37.6%) | 0.027 | 181/364  (49.7%) | 31/94 (33%) | 0.004 | 199/405  (49.1%) | 13/53  (24.5%) | <0.001 |
| Long-term health-related quality of life | | | | | | | | | | |
| Total | 6 (5-9) | 6 (5–8) | 8 (6–11) | 0.001 | 6 (5-8) | 9 (6-12) | <0.001 | 6 (5-9) | 9.5 (5-12) | 0.067 |
| Mobility | 1 (1-2) | 1 (1–2) | 2 (1–3) | 0.024 | 1 (1-2) | 2 (1-3) | 0.002 | 1 (1-2) | 2 (1-3) | 0.10 |
| Self-care | 1 (1-2) | 1 (1–1) | 1 (1–3) | <0.001 | 1 (1-2) | 2 (1-3) | <0.001 | 1 (1-2) | 2 (1-3) | 0.016 |
| Usual activities | 1 (1-2) | 1 (1–2) | 1 (1–3) | <0.001 | 1 (1-2) | 2.5 (1-3) | <0.001 | 1 (1-2) | 3 (1-3) | 0.031 |
| Pain/discomfort | 1 (1-2) | 1 (1–2) | 1 (1–2) | 0.12 | 1 (1-2) | 1 (1-2) | 0.35 | 1 (1-2) | 3 (1-3) | 0.63 |
| Anxiety/depression | 1 (1-1) | 1 (1–1) | 1 (1–2) | 0.23 | 1 (1-1) | 1 (1-2) | 0.35 | 1 (1-1) | 1 (1-2) | 0.13 |
| EQ-5D utility index (median, IQR) | 0 (0-0.9) | 0.55 (0–0.94) | 0 (0–0.70) | <0.001 | 0.55 (0-0.95) | 0 (0-0.64) | <0.001 | 0.55 (0-0.92) | 0 (0-0.49) | <0.001 |
| eTICI, expanded treatment in cerebral infarction; IQR, interquartile range; mRS, modified Rankin Scale; EQ-5D3L, EuroQol Group 5-dimension 3-level; EQ-5D, EuroQol Group 5-Dimension. | | | | | | | | | | |
