# Supplementary material for: Long-Term Effect of Mechanical Thrombectomy in Stroke Patients According to Advanced Imaging Characteristics
Source: Clin Neuroradiol. 2023 Aug 29;34(1):105–14. doi: 10.1007/s00062-023-01337-4 (PMC10881753; doi:10.1007/s00062-023-01337-4)
Supplement: Supplementary file 2 — STROBE Checklist [file 62_2023_1337_MOESM2_ESM.doc]

STROBE Statement—Checklist of items that should be included in reports of ***cohort studies***

**Long-Term Effect of Mechanical Thrombectomy in Stroke Patients according to Advanced Imaging Characteristics. Beyeler et al.**

|  | Item No | Recommendation |
| --- | --- | --- |
| **Title and abstract** | 1 | (*a*) Indicate the study’s design with a commonly used term in the title or the abstract  🡪 Retrospective, monocentric and cohort study in abstract (p.1) |
|  |  | (*b*) Provide in the abstract an informative and balanced summary of what was done and what was found  🡪 Available (p.1-2) |
| Introduction | | |
| Background/rationale | 2 | Explain the scientific background and rationale for the investigation being reported  🡪 Available (p.3) |
| Objectives | 3 | State specific objectives, including any prespecified hypotheses  🡪 Available (p.3) |
| Methods | | |
| Study design | 4 | Present key elements of study design early in the paper  🡪 Available (p.4) |
| Setting | 5 | Describe the setting, locations, and relevant dates, including periods of recruitment, exposure, follow-up, and data collection  🡪 Available (p.4-5) |
| Participants | 6 | (*a*) Give the eligibility criteria, and the sources and methods of selection of participants. Describe methods of follow-up  🡪 Available (p.4-5) |
|  |  | (*b*) For matched studies, give matching criteria and number of exposed and unexposed  🡪 Not applicable |
| Variables | 7 | Clearly define all outcomes, exposures, predictors, potential confounders, and effect modifiers. Give diagnostic criteria, if applicable  🡪 Available (p. 4-6) |
| Data sources/ measurement | 8* | For each variable of interest, give sources of data and details of methods of assessment (measurement). Describe comparability of assessment methods if there is more than one group  🡪 Available (p. 4-6) |
| Bias | 9 | Describe any efforts to address potential sources of bias  🡪 Done, inclusion of all deceased patients following the ethics committee’s decision. |
| Study size | 10 | Explain how the study size was arrived at  🡪 Not applicable: Cohort study including all consecutive patients treated with mechanical thrombectomy between 2010-2018 |
| Quantitative variables | 11 | Explain how quantitative variables were handled in the analyses. If applicable, describe which groupings were chosen and why  🡪 Available (p. 4-6) |
| Statistical methods | 12 | (*a*) Describe all statistical methods, including those used to control for confounding  🡪 Available (p.5-6) |
|  |  | (*b*) Describe any methods used to examine subgroups and interactions  🡪 Available (p.6) |
|  |  | (*c*) Explain how missing data were addressed 🡪 Available (p.6) |
|  |  | (*d*) If applicable, explain how loss to follow-up was addressed 🡪 Loss to follow-up were excluded as mentioned (p.4) |
|  |  | (*e*) Describe any sensitivity analyses 🡪 No sensitivity analyses per se |
| Results | | |
| Participants | 13* | (a) Report numbers of individuals at each stage of study—eg numbers potentially eligible, examined for eligibility, confirmed eligible, included in the study, completing follow-up, and analysed 🡪 Available (summarised in eFigure 1) |
|  |  | (b) Give reasons for non-participation at each stage 🡪 eFigure 1 |
|  |  | (c) Consider use of a flow diagram 🡪 eFigure 1 |
| Descriptive data | 14* | (a) Give characteristics of study participants (eg demographic, clinical, social) and information on exposures and potential confounders 🡪 Available (eTable 1 and Table 1) |
|  |  | (b) Indicate number of participants with missing data for each variable of interest  🡪 Available in Tables. |
|  |  | (c) Summarise follow-up time (eg, average and total amount) 🡪 Available (Table 1 and p.8) |
| Outcome data | 15* | Report numbers of outcome events or summary measures over time 🡪 Available (p.7-8) |
| Main results | 16 | (*a*) Give unadjusted estimates and, if applicable, confounder-adjusted estimates and their precision (eg, 95% confidence interval). Make clear which confounders were adjusted for and why they were included 🡪 Unadjusted baseline characteristics available, for the regression analysis only multivariate analysis reported in the paper. (p.8-9) |
|  |  | (*b*) Report category boundaries when continuous variables were categorized  🡪 Available (p.8-9) |
|  |  | (*c*) If relevant, consider translating estimates of relative risk into absolute risk for a meaningful time period 🡪 Not relevant |
| Other analyses | 17 | Report other analyses done—eg analyses of subgroups and interactions, and sensitivity analyses 🡪 Available (p.8-9) |
| Discussion | | |
| Key results | 18 | Summarise key results with reference to study objectives 🡪 Available (p.9) |
| Limitations | 19 | Discuss limitations of the study, taking into account sources of potential bias or imprecision. Discuss both direction and magnitude of any potential bias  🡪 Available (p.11) |
| Interpretation | 20 | Give a cautious overall interpretation of results considering objectives, limitations, multiplicity of analyses, results from similar studies, and other relevant evidence  🡪 Available (p.9-11) |
| Generalisability | 21 | Discuss the generalisability (external validity) of the study results  🡪 Available (p.9-11) |
| Other information | | |
| Funding | 22 | Give the source of funding and the role of the funders for the present study and, if applicable, for the original study on which the present article is based  🡪 Available (p.13) |
